# Apology and Its Acceptance: Perceived Reconciliatory Attitudes Reduce Outgroup Dehumanization

**DOI:** 10.3389/fpsyg.2022.809513

**Published:** 2022-04-25

**Authors:** Wen Jie Jin, Sang Hee Park, Joonha Park

**Affiliations:** ^1^Department of Psychology, Chungbuk National University, Cheongju, South Korea; ^2^Graduate School of Management, Nagoya University of Commerce and Business, Nagoya, Japan

**Keywords:** apology, acceptance, human nature, human uniqueness, historical conflict, intergroup relations

## Abstract

Based on real-life intergroup animosities originating from a historical conflict, the current study examined how the perceived stance of the outgroup about the conflict affects the dehumanization of the outgroup. In Study 1 (*N* = 120), Korean undergraduates attributed more *human nature* to the Japanese after reading an article that the Japanese government did (vs. refused to) issue an official apology for a historical wrong. In turn, the more human nature assigned to the Japanese predicted higher expectations about positive mutual relations in the future. Similarly, in Study 2 (*N* = 209), Japanese undergraduates attributed more *human uniqueness* to Koreans after reading an article that an official apology for a historical wrong from Japan was accepted (vs. rejected) by Koreans. The higher the perceived human uniqueness of Koreans was, the higher were the willingness to help and the expectations of a positive relationship in the future. The findings demonstrate how mutual dehumanization can be reduced as a result of the other side’s reconciliatory stances and can further contribute to improving intergroup relations.

## Introduction

Intergroup conflicts often leave behind lasting feelings of animosity and invisible intergroup tension between groups even after conflicts seem to have been dissolved by glance. In international relations, the conflict between Israel and Palestine has a prolonged and bitter history, and the relationship between Korea and Japan has long been in a persisting impasse over the issue of Japanese military sexual slavery during WWII (Piper, 2001; Hayashi, 2008). Unfortunately, these conflicts become obstacles to the peaceful development of relations between the countries involved. Dehumanization or denial of human qualities (Vaes et al., 2003; Castano and Giner-Sorolla, 2006; Haslam, 2006; Goff et al., 2008; Maoz and McCauley, 2008; Haque and Waytz, 2012) is often observed in the context of intergroup conflict as a precursor to harmdoing. While the outright denial of humanness may occur only in extreme cases of antagonism such as genocides (Kelman, 1976; Chalk and Jonassohn, 1990), research has shown that there are also more subtle forms of dehumanization observed as slightly less ascription of human characteristics (Haslam, 2006; Bastian and Haslam, 2011). Even though these more “everyday” kinds of dehumanization may seem trivial and harmless, they reflect the ways that conditions of conflict can shape the groups’ perception of each other and can uniquely predict various outcomes such as reduced intergroup helpfulness (Cuddy et al., 2007) and increased aggressiveness (Viki et al., 2013).

In the current research, we examined how two groups in a conflict perceive each other’s humanness differently as a function of the ingroup’s status in the conflict (the victim vs. the perpetrator) and the other group’s stance on reconciliation. We studied this question in the context of a historical conflict between Korea and Japan (i.e., the issues of Japanese sexual slavery and forced labor; Hayashi, 2008). We hypothesized that, when the other group makes (vs. rejects to make) a move toward reconciliation, members of historically victimized and perpetrating groups would be more likely to perceive different aspects of humanness in each other (Haslam, 2006). In turn, such a higher perception of the other’s human qualities would lead to more amicable intentions toward the other and to more optimistic expectations about future intergroup relationships. Thus, the current research examined how the images of the adversary can become more human in distinct ways as a result of the adversary’s reconciliatory gestures.

### Humanness Perception and Dehumanization

Oftentimes, individuals and group members are perceived as lacking in humanness, especially when they are associated with negativity (Leyens et al., 2000, 2001; Haslam et al., 2005; Haslam, 2006; Bain et al., 2009; Bastian and Haslam, 2011; Waytz and Epley, 2012; Haslam and Loughnan, 2014; Park and Park, 2015). According to Haslam (2006) model of dehumanization, there are two distinct dimensions of humanness that are largely independent of each other (Haslam et al., 2005; Haslam, 2006). One dimension, labeled human nature (HN), is a group of traits that differentiate humans from machines and inanimate objects. Collectively these reflect emotional responsiveness and warmth, and include traits such as “emotional,” “optimistic,” and “curious.” The other dimension is human uniqueness (HU), which is a set of characteristics that distinguish human beings from other animals. HU represents civility, rationality, capability, and moral sensibility and can include traits like “assertive,” “self-controlled,” and “humble.” According to Haslam (2006), failing to recognize a person’s HN will result in the person being seen as cold, rigid, inert, passive, superficial, and unemotional, like a machine (*mechanistic dehumanization*), while ignoring a person’s HU will lead to perceiving the person as irrational, uncultured, amoral, childlike, coarse, and out of control, like a non-human animal (*animalistic dehumanization*). These two distinct forms of dehumanization have been observed in implicit perceptions of different social categories, such as artists and businesspeople (Loughnan and Haslam, 2007), the elderly (Boudjemadi et al., 2017), professionals, and the so-called “lowest of the low” (e.g., drug addicts and homeless people; Morera et al., 2018).

Research has documented the various adverse impact of dehumanization: those who dehumanize others show reduced willingness to help the targets (Cuddy et al., 2007; Viki et al., 2012); give harsher punishment to the targets (Viki et al., 2013); and also behave more aggressively toward the targets (Bandura et al., 1975; Greitemeyer and McLatchie, 2011). For example, Viki et al. (2013) showed that Christians were more likely to recommend the torture of dehumanized Muslim prisoners when they read vignettes describing low (vs. high) humanity of Muslim prisoners.

In light of these findings, to avoid further deterioration of intergroup relations, it is imperative to identify the conditions under which parties in an intergroup conflict are denied human qualities. To achieve this aim, we need to first consider the motivations for dehumanizing others; in other words, the functions that dehumanization serves in the intergroup context need to be considered.

### Motivations for Mutual Dehumanization in Intergroup Conflicts

Is dehumanization toward an outgroup a privilege that only the advantaged group can possess (Kelman, 1976; Chalk and Jonassohn, 1990; Castano and Giner-Sorolla, 2006)? Showing that the powerless can also dehumanize the powerful, Kteily and Bruneau (2017) demonstrated that two parties (i.e., Israelis and Palestinians) with asymmetric power in a conflictual relationship engaged in blatant dehumanization of each other. Interpreting these results using Haslam (2006) dehumanization theory, people who are disadvantaged in conflict situations will experience more serious damage, so they would perceive the advantaged group as machines that cannot feel emotions. On the other hand, the advantaged group can perceive the disadvantaged group to be inferior to humans to address moral threats derived from their offensive actions.

Along similar lines, studies have provided evidence of mutual dehumanization between the perpetrator and the victim group in conflict contexts. Specifically, previous research demonstrated cases in which the victim perceives less HN in the perpetrator. For example, Leidner et al. (2013, Study 1) observed that Palestinians (victims) were less likely to perceive Israelis’ (perpetrators’) sentience to feel and experience emotions (i.e., HN). Also, the more Palestinians mechanically dehumanized Israelis, the higher was their willingness to punish Israelis. Similarly, Bastian and Haslam (2010) found in the context of interpersonal ostracism that, when participants were socially excluded, they were more likely to deny the perpetrator’s HN. In contrast, the perpetrator may perceive less HU in the victim. Castano and Giner-Sorolla (2006) demonstrated that reminding participants of their ingroup’s harmdoing in the past can make them deny the victim group members’ capabilities of experiencing secondary emotions (Leyens et al., 2000). For example, in Experiment 3 (Castano and Giner-Sorolla, 2006), European American participants were less likely to attribute secondary emotions to Native Americans when they had to justify their ingroup’s wrongdoings (i.e., historical persecution of Native Americans), indicating denial of HU in the victim group members. These findings indicate that the victim group and the perpetrator group can dehumanize the other party in different ways.

### Approaches to Promoting Conflict Resolution: Apology and Apology Acceptance

Among various approaches to reducing intergroup dehumanization, the current study focuses on the effects of apology (by the perpetrator group) and the acceptance of apology (by the victim group). Both of the conditions can contribute to the improvement of intergroup relations (Shnabel and Nadler, 2008). For example, in Brown et al. (2008), members of a victim group reported greater willingness to forgive and reconcile when they received an apology from the perpetrator group (see also Shnabel et al., 2009). Borinca et al. (2021, Study 3) demonstrated that an apology offered by the perpetrator group (i.e., Serbians) reduced victims’ (i.e., Albanians) dehumanization of them more than when the apology was refused by the perpetrator group, and then, such lessened dehumanization facilitated the victims’ willingness to intergroup contact and reconciliation. In a study by Vaes and Bastian (2021, Study 4), a victim who suffered social exclusion exhibited less dehumanization and more intention to forgive the perpetrator when they received an apology than when they did not receive it from the perpetrator. According to Leidner et al. (2013, Study 1), if the victims receive apologies from the perpetrators, they would be more likely to reappraise the perceived sentience of the perpetrators and to perceive their HN. On the other hand, when perpetrators’ apology was accepted rather than rejected by the victim group, the perpetrators showed more positive attitudes toward the victims and stronger intentions for reconciliatory actions (e.g., willingness to compensate; Harth et al., 2011). For instance, according to Vaes and Bastian (2021, Study 3), a perpetrator was less willing to dehumanize the victim when the apology for wrongdoing (i.e., treating the victim rudely) was accepted vs. rejected by the victim. Considering that the need to justify that the ingroup’s wrongdoings instigated the perpetrators’ dehumanization of the victim group (Castano and Giner-Sorolla, 2006), the victims’ acceptance of the perpetrators’ apologies may relieve perpetrators of their collective self-threat and guilt, resulting in increased perception of HU in the victim group members.

Similar to the current study, Borinca et al. (2021, Study 3) investigated the effect of the perpetrator group’s apology on the improvement of intergroup relations through the victims’ perception of humanness, and Vaes and Bastian (2021, Study 3) investigated whether accepting an apology or not affects dehumanization toward the perpetrator. However, Borinca and colleagues only focused on the position of the victim group members and did not verify the impact of intergroup apology on specific dimensions of humanness, and Vaes and Bastian primarily focused on interpersonal rather than intergroup conflict. Our research takes a broader and specific perspective to examine the role of apology and acceptance of apology on mutual dehumanization of both sides in conflicts as well as their downstream effects on the dynamics of intergroup relations.

### The Present Research

The purpose of this research is to explore patterns of mutual dehumanization between two groups that have a history of being victims and perpetrators. Specifically, we asked Korean (historical victims; Study 1) and Japanese (historical perpetrators; Study 2) participants to read an article describing that the other nation made a gesture that is either reconciliatory (i.e., Japanese apologized or Koreans accepted Japan’s apology) or non-reconciliatory (i.e., Japanese refused to apologize or Koreans refused to accept the outgroup’s apology), and observed how it affected the way participants assign human qualities to the other group. We hypothesized that, when the gesture is reconciliatory (vs. non-reconciliatory), participants would perceive the other group as more human. In both groups, because there is less need to explain and justify one’s position by derogating the other, there would be less reason to dehumanize them. Therefore, it was predicted that Korean participants would assign more HN to Japanese when they read the article that the Japanese issued an apology, and that Japanese participants would assign more HU to Koreans when they read the article that Koreans accepted Japan’s apology. Many previous studies have shown that dehumanization can occur in intergroup conflicts (Castano and Giner-Sorolla, 2006; Viki et al., 2013), resulting in increased aggressiveness (Bastian et al., 2012; Viki et al., 2013). It is suggested that an apology can alleviate such negative consequences (Leonard et al., 2011; Wenzel et al., 2017), and that forgiveness can relieve negative sentiments caused by intergroup conflicts (Wenzel and Okimoto, 2015). However, research has rarely investigated the direct consequences of apology or apology acceptance on humanness attribution, which is the main focus of the current study.

We also aimed to test downstream consequences of humanness attributions on the outlook of future intergroup relationships. Tam et al. (2007) demonstrated how degrees of perceived outgroup humanness (operationalized as infrahumanization), along with anger and attitude toward the outgroup, can predict intergroup forgiveness. However, their correlational studies only looked at a single variable of intergroup forgiveness as an outcome of contact. In the current investigation, we looked at whether apology-related manipulations (apology in Study 1 and apology acceptance in Study 2) can predict various relationship outcome variables, mediated by attributions of humanness to the outgroup.

## Study 1

In Study 1, we examined whether Koreans would perceive Japanese people’s humanness differently depending on their current stance regarding the historical conflict. We also examined the effects of the Japanese apology on the Koreans’ thoughts about Korean-Japanese relations. In addition to a willingness to forgive, other variables that are highly relevant to conflict resolution between formerly adversary countries (i.e., willingness to help, expectation of a future relationship) were also measured (Andrighetto et al., 2014). To explore our research questions, we constructed three fictitious articles: In two, the Japanese government and its citizens were described as being apologetic or not, and in the last, neutral information about Japan was given. Specifically, we used the issue of WWII sexual slavery by the Japanese military as the context of intergroup conflict and presented to Korean participants—the victim group members—the information that the government and civilians of Japan had apologized or refused to apologize for this crime (apology issued vs. apology refused condition). Ninety-six percent of South Koreans regard themselves as vicarious victims of the Japanese occupation in the early twentieth century and think that the Japanese government should have apologized for its past atrocities (Chun, 2015). Although there actually have been issuances of apology by Japanese officials and civilians in the past (e.g., Murayama Statement in 1995), Koreans in general have been viewing them with skepticism because of a lack of apparent authenticity, especially in light of contradictory remarks and behaviors of Japanese high-profile officials (Hayashi, 2008). This provides an ideal context for testing the effects of intergroup apology because it is possible to manipulate the perception of whether the Japanese expressed an apology or refused to without compromising its believability. We expected that (a) when the Japanese were described as apologetic (vs. unapologetic) of the historical victimization of Koreans, Korean participants would attribute more HN to the Japanese (Bastian and Haslam, 2010; Leidner et al., 2013), and that (b) this attribution of HN would explain intentions and expectations about the future intergroup relationship. We did not make any specific predictions regarding the control condition because it was unclear how the results would turn out when there is no mention of the historical issues.

### Materials and Methods

#### Participants

One hundred and thirty-three undergraduate students from a large public university in Korea taking psychology courses participated for course credit. Nine participants who failed the attention checks were excluded, as well as 3 participants who were not of Korean nationality. Data from one participant were lost because of a computer malfunctioning. Thus, there were 120 participants in the final sample (54 females, age *M* = 21.63, *SD* = 2.26). The sample size was not determined by an *a priori* power analysis. A sensitivity analysis using G*Power 3.1.9.2 (Faul et al., 2007) found that our final sample size was sufficient to detect a minimum detectable effect size of *f* = 0.29 with 80% power and alpha of 0.05 across the three conditions. This study was approved by the university’s Institutional Review Board (IRB). We collected as many participants as possible in an entire semester in both studies.

#### Manipulation

Participants were randomly assigned to one of three conditions. In the apology issued condition (*n* = 39), participants read an article about the sexual slavery of Korean women by the Japanese military in World War II (so-called “comfort women”), followed by a statement that Japanese citizens and government officials have apologized for this wrongdoing. The essential elements of intergroup apology were used to construct this article.^1^ To strengthen the manipulation effect, the article also reported the results of a fictitious recent survey that the majority of Japanese people thought that it was necessary for the Japanese government to officially apologize. In the apology refused condition (*n* = 38), the article was modified so that the Japanese government was described as having refused to acknowledge their responsibilities for the sexual slavery issue. We also presented a fictitious survey report that described Japanese public opinion as mostly against apologizing to Korea. Participants in the control condition (*n* = 43) read an article about the climate of Japan. We set the control condition with an issue irrelevant to the conflict because of the concern that participants could automatically associate the Korea–Japan historic conflict with Japanese’s unapologetic attitudes as usual (Oh and Shin, 2010).

#### Measures

All measures used 7-point Likert scales (ranging from 1 *strongly disagree* to 7 *strongly agree*), except for the attention check, which consisted of two yes/no questions. First, participants in all conditions were asked to evaluate the humanness of the people of the two countries and complete other exploratory measures (e.g., perceived moral superiority of Koreans over Japanese, perceived sincerity of the issued apology). No results of interest were observed and these were not discussed further. Next, they rated relationship outcome variables (willingness to help Japan in need, willingness to forgive Japan’s past wrongdoings, and expectation of a future relationship with Japan), followed by manipulation check and attention check measures. We only reported results for the Korean participants’ perception of the Japanese, which was of our primary interest. All reported items are listed in Supplementary Material.

#### Perceived Humanness

The perceived humanness of the two groups (Korean and Japanese people) was measured in two ways as in previous research (Bastian and Haslam, 2010; Bastian et al., 2012; Yang et al., 2015): agreement with statements describing either HN (e.g., “I can feel warmth in Koreans/Japanese”) or HU (e.g., “I think Koreans/Japanese are refined and cultured”) as well as ratings on traits relevant to HN or HU. First, twelve humanness statements were adapted from Bastian and Haslam (2010) Study 2 for our target groups. Following previous studies on dehumanization (e.g., Bastian and Haslam, 2010; Bastian et al., 2012; Pacilli et al., 2016), indices of HN and HU for the two targets were obtained by reversing scores of the low HN/HU and averaging all the scores for each dimension (see Table 1 for internal consistency indices) so that higher scores mean lower levels of dehumanization. Second, trait measures were constructed using 16 personality traits selected from an original pool of 40 traits varying in HN and HU (J. Park et al., 2012). However, because some of the reliability scores for trait humanness were extremely low in the present studies (αs = 0.27–0.67), we dropped the results obtained with trait measures and only the results using the statement humanness measures are reported.

**TABLE 1:** Reliability indices and correlations among measured variables (Study 1, *N* = 120).

	α	1	2	3	4	5
1. Japanese HN statements	0.785	–	0.515**	0.323**	0.281**	0.402**
2. Japanese HU statements	0.681		–	0.299**	0.252**	0.456**
3. Willingness to help	0.955			–	0.197*	0.499**
4. Willingness to forgive	0.822				–	0.356**
5. Expectation of a future relationship	0.819					–

*p < 0.05, **p < 0.01.

#### Relationship Outcome Variables: Willingness to Help, Willingness to Forgive, and Expectation of a Future Relationship

Questions on willingness to help (3 items; e.g., “If many people in Japan were to die because of an earthquake, we should help them”), willingness to forgive (4 items; e.g., “Koreans may be able to forgive the wrongdoings that Japan has perpetrated in the past”), and expectation of a future relationship (4 items; e.g., “The relationship between Korea and Japan will be better than now”) were administered to tap prospective attitudes toward the outgroup (see Table 1 for reliabilities). Participants also completed manipulation check (2 items, *r* = 0.83; e.g., “Japan seems to have acknowledged its past wrongdoings against Korea”) and attention check (2 items; e.g., “According to this article, Japanese government acknowledged the forced recruitment of comfort women”) measures.

#### Procedure

After participants arrived at the laboratory, they were greeted by the experimenter and were told that they would participate in an experiment about “how people perceive other countries.” The study was conducted on the computer using Inquisit software (Inquisit 4, 2015). After reading one of the articles, participants completed a set of questionnaires and provided demographic information (gender, age, and nationality), made a guess about the study’s real purpose, and then were debriefed before leaving the room. No participant suspected the study’s true purpose.

### Results

Correlations among the measured variables are reported in Table 1, and the means and standard deviations by conditions are presented in Table 2.

**TABLE 2:** Means (standard deviations) of variables in the three experimental conditions (Study 1).

	Condition		
	Apology issued	Apology refused	Control
Manipulation check	4.01_a_ (1.43)	1.76_b_ (0.86)	1.94_b_ (0.74)
Perceived humanness of Japanese			
HN statements	4.46_a_ (1.01)	3.82_b_ (1.02)	4.61_a_ (0.65)
HU statements	4.28_ab_ (0.77)	4.09_a_ (0.83)	4.62_b_ (0.71)
Relationship outcome measures			
Willingness to help	4.85 (1.46)	4.93 (1.67)	5.09 (1.27)
Willingness to forgive	3.26 (1.49)	3.19 (1.34)	2.78 (1.19)
Expectation of a future relationship	4.60 (0.92)	4.36 (0.91)	4.55 (0.95)

*Mean values in a row with different subscripts are significantly different at p < 0.05 using Tukey’s HSD post hoc test, and no subscripts indicate no significant differences between the conditions.*

#### Manipulation Check

The condition effect on the manipulation check measure was significant, *F*(2, 117) = 56.06, *p* < 0.001, η^2^ = 0.49. *Post hoc* comparisons (Tukey’s HSD) showed that perceived apology was greater in the apology issued condition than in the other two conditions [vs. the apology refused condition, *p* < 0.001, 95% CI (1.68, 2.82), and vs. the control condition, *p* < 0.001, 95% CI (1.52, 2.62)]. There was no difference between the latter two conditions, *p* = 0.724, 95% CI (–0.37, 0.37).

#### Humanness Attributions

For the perceived humanness of Japanese people, a set of one-way ANOVAs were conducted on HN and HU (Table 2). There was a significant condition effect on HN, *F*(2, 117) = 8.46, *p* < 0.001, η^2^ = 0.13. *Post hoc* comparisons showed that HN were more endorsed in the apology issued condition than in the apology refused condition, *p* = 0.007, 95% CI (0.15, 1.13). Participants also attributed more HN to the Japanese in the control condition than in the apology refused condition, *p* < 0.001, 95% CI (0.31, 1.27). There was no difference between the apology issued and the control conditions, *p* = 0.730, 95% CI (–0.63, 0.32). These results lend support to our hypothesis that Korean participants would view Japanese as being higher on HN when the Japanese were perceived as being apologetic (vs. unapologetic).

Additionally, the endorsement of HU differed by condition, *F*(2, 117) = 4.94, *p* = 0.009, η^2^ = 0.08. Compared to the control condition, HU were less endorsed in the apology refused condition, *p* = 0.007, 95% CI (–0.93, –0.12). There was no difference between the apology issued and the apology refused conditions, *p* = 0.526, 95% CI (–0.23, 0.61) or between the apology issued and the control conditions, *p* = 0.119, 95% CI (–0.74, 0.07) on the ratings of HU.

#### Relationship Outcome Measures

Overall, participants in all conditions showed less willingness to forgive compared to the midpoint, *t*(119) = –7.59, *p* < 0.001. However, their willingness to help Japanese in need and expected future positive relationship were both higher than the midpoint, *t*(119) = 7.19, *t*(119) = 5.96, respectively, both *p*s < 0.001. Contrary to our prediction, there were no significant differences across conditions on the willingness to help, *F*(2, 117) = 0.28, *p* = 0.755, the willingness to forgive, *F*(2, 117) = 1.52, *p* = 0.223, or the expectation of a future relationship, *F*(2, 117) = 0.68, *p* = 0.508. Thus, apology from the outgroup (perpetrators) seems to have little effects on the participant’s willingness or expectation about relationship improvement.

#### Mediation Analysis

Although we did not see significant effects of apology on any of the relationship outcome measures, the result does not necessarily preclude us from testing the hypothesized mediation-by-dehumanization model. That is, while the traditional understanding of mediation (e.g., Baron and Kenny, 1986) requires that there be a significant zero-order relationship between the independent and the dependent variables before a mediation model is tested, more recent statistical developments showed that such a precondition does not have to be met (Hayes, 2009; Zhao et al., 2010; Rucker et al., 2011). In our case, given the significant effect of apology on the perception of HN in Japanese as well as the correlations between HN and relationship outcome measures (Table 1), it is highly probable that HN would mediate the relationship between apology manipulation and relationship outcome measures.

**FIGURE 1:**
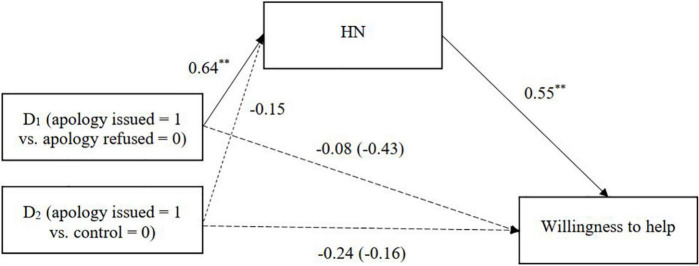
HN mediating the relationship between condition and willingness to help (Study 1). Unstandardized coefficients are presented. Coefficients in parentheses denote direct effects after controlling for indirect effects. ^**^p < 0.01.

**FIGURE 2:**
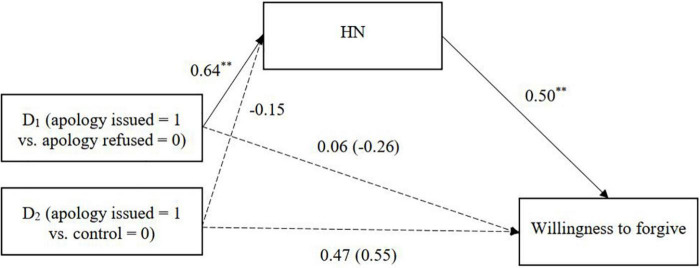
HN mediating the relationship between condition and willingness to forgive (Study 1). Unstandardized coefficients are presented. Coefficients in parentheses denote direct effects after controlling for indirect effects. ^**^p < 0.01.

**FIGURE 3:**
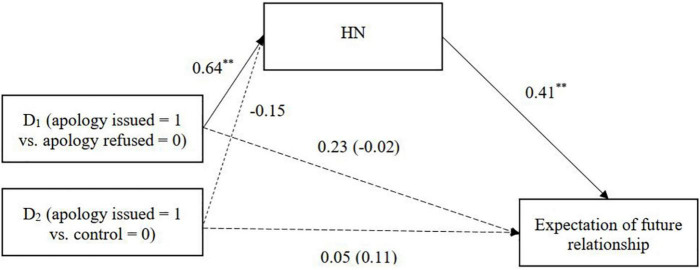
HN mediating the relationship between condition and expectation of a future relationship (Study 1). Unstandardized coefficients are presented. Coefficients in parentheses denote direct effects after controlling for indirect effects. ^**^p < 0.01.

To test this, we conducted a series of multi-categorical mediation analyses using the bootstrapping procedure of SPSS PROCESS macro (Model 4; Hayes and Preacher, 2014) with 5,000 resamplings. The relative indirect effects were estimated by comparing each condition (apology refused and control) with the apology issued condition. For the multicategorical independent variables (3 conditions), the program automatically generated two dummy variables with the apology issued condition as a reference: one that contrasts with the apology refused condition and the other that contrasts with the control condition. Significant indirect effects of the condition through HN on all three outcome measures were observed (Table 3 and Figures 1–3). Korean participants attributed more HN to the Japanese when the apology was issued (vs. refused), and the HN attributed toward the Japanese in turn was associated with stronger intentions to help and forgive the outgroup and anticipations of future intergroup relations.

**TABLE 3:** Indirect effects of condition on relationship outcome measures through human nature (HN) (Study 1).

	Condition → HN → willingness to help	Condition → HN → willingness to forgive	Condition → HN → expectation of a future relationship
Apology issued (1) vs. apology refused (0)	0.35 [0.08, 0.66]	0.32 [0.08, 0.61]	0.26 [0.07, 0.48]
Apology issued (1) vs. control (0)	–0.08 [–0.32, 0.12]	–0.08 [–0.29, 0.12]	–0.06 [–0.24, 0.09]

*Numbers indicate unstandardized regression coefficients for indirect effects and respective 95% CIs.*

### Discussion

In Study 1, Korean participants attributed more HN to Japanese people when the Japanese were described as being apologetic for their ingroup’s past wrongdoings compared to when they were described as refusing to apologize. The varied perception of humanness in perpetrators by the victim group members was concentrated on HN, in line with our assumption that what is regarded as redeemed through apology are mainly the aspects of humanness that set people apart from unemotional, machine-like beings (Haslam, 2006; Bastian and Haslam, 2010).

Whether or not the Japanese apologized to Koreans did not significantly affect any relationship outcome measures. Interestingly though, there were mediation effects: When Japanese attitudes were described as apologetic toward Koreans, the participants attributed more HN to the outgroup, which in turn predicted more willingness to help, more willingness to forgive, and more positive expectations of a future relationships. The lack of direct condition effects on relationship measures is consistent with previous findings (e.g., Philpot and Hornsey, 2008, 2011; Wohl et al., 2012) and may hint at unexplained suppression effects (MacKinnon et al., 2000; Rucker et al., 2011). For example, some participants in the apology issued condition might have felt unsatisfied with the perpetrator’s apology (Philpot and Hornsey, 2008) or regarded it as not a genuine victim-focused apology but just a formalism (Berndsen et al., 2015; Chun, 2015). Still, the present results indicate that under certain circumstances, reconciliatory gestures from the offending group can increase their HN in the victim group members’ eyes, which is positively associated with the victim group members’ willingness for a more positive future relationship.

In sum, Study 1 showed that members of a historically victimized group may attribute HN to the perpetrator group to a different degree, contingent on whether the perpetrator group apologized or not, and that such perceived HN in turn predicts their outlook on future intergroup relations. Switching to the other side of the historical conflict, Study 2 explored how the perpetrator group members would differently perceive the victim group members’ HU depending on the victim group’s acceptance of their apology.

## Study 2

As a parallel to Study 1, we next examined how the victim group’s (Koreans’) acceptance (vs. rejection) of the perpetrator group’s (Japanese’) apology would affect the perpetrator group’s perception of HU in the victim group. We also examined the effects of apology acceptance on Japanese’s attitudes and expectations of the future intergroup relationship. In this frame, modern-day Japanese people are considered as the perpetrator group’s representatives, as they may experience group-based guilt for the past based on group membership (e.g., Branscombe et al., 2002). We constructed three articles based on a similar controversy to the “comfort women” issue in Study 1, namely the forced labor abuses of Korean workers committed by Japanese companies during WWII. Along with the “comfort women,” Japanese private companies’ involvement in forced wartime labors of Koreans during the colonization period has been another sensitive issue in both societies, particularly in Japan. Causing tensions between the two nations, the solutions for compensation remain incomplete in many cases (Soble, 2013). However, it is a relatively less known issue to most Japanese (Shibata, 2018) and thus is a suitable context in which to manipulate Koreans’ acceptance or rejection of Japanese apology. If the Japanese learn that Koreans accept the ingroup’s apology, they would release the need to justify the past wrongdoing and thus find less reason to derogate the outgroup in terms of HU. Therefore, we predicted that Japanese participants would attribute Koreans more HU when they learned of Koreans’ acceptance (vs. rejection) of the ingroup apology. We also expected that the difference in HU attribution would explain intentions and expectations about future intergroup relationships. As in Study 1, we left the question of what would happen in the control condition, where only materials irrelevant to the forced labor issue were presented openly.

### Materials and Methods

#### Participants

Two hundred and nine Japanese undergraduate students (38 females, age *M* = 19.17, *SD* = 1.35) at a Japanese private university participated in a paper-and-pencil survey for course credits in lecture settings. The sample size was not determined by an *a priori* power analysis. A sensitivity analysis conducted using G*Power 3.1.9.2 (Faul et al., 2007) showed that the current sample size was sufficient to detect an effect with a minimum detectable effect size of *f* = 0.22 with 80% power and alpha of 0.05 across the three conditions. Because one participant did not fill out responses to all of the four items related to the expectation of future relationships, his data were excluded from all analyses that included the computed variable of those items. The study was approved by the university IRB.

#### Manipulation

As in Study 1, participants were randomly assigned to one of three conditions. Participants first read an article in which the outgroup’s apology acceptance was manipulated. The article in the apology accepted condition (*n* = 74) described a fictitious survey in South Korea reporting that the Korean government and the people showed satisfaction and willingness to accept the recent apology of a Japanese company, whereas the article in the apology rejected the condition (*n* = 66) that described a fictitious survey that Koreans did not show satisfaction and willingness to accept the company’s recent apology. The article in the control condition (*n* = 69) described temperatures over four seasons in the Korean peninsula. In order to block unintentional effects on outgroup evaluation, similar to Study 1, the control condition did not include any information that may evoke participants’ awareness of intergroup conflicts or the issue of the apology.

#### Measures

The measures administered after the article were largely identical to those in Study 1, except that the items asked about the Japanese participants’ perceptions of Koreans and what they think of the relations with Korea. Also, instead of a willingness to forgive, participants were asked to indicate a willingness to apologize (e.g., “Japan could apologize if it had done something wrong with Korea in the past.”) for the past historical faults. Correlations among main variables and inter-item reliabilities for each variable are reported in Table 4.

**TABLE 4:** Reliability indices and correlations between main variables (Study 2, *N* = 209).

	α	1	2	3	4	5
1. Korean HN statements	0.773	–	0.564**	0.105*	0.262**	0.285**
2. Korean HU statements	0.752		–	0.196**	0.126	0.335^***^
3. Willingness to help	0.928			–	0.185**	0.222**
4. Willingness to apologize	0.652				–	0.091
5. Expectation of a future relationship	0.944					–

*p < 0.05,**p < 0.01,**p < 0.001.

#### Procedure

As in Study 1, after reading a randomly assigned article, participants were asked to evaluate the humanness of each of the two national groups (the Japanese and Koreans in order) on a set of 16 personality traits and 12 statements. Expectation of a future relationship between the two countries and other exploratory variables (justification of the historical fault of Japanese toward Koreans, economic and moral statuses of Japan and Korea) were also measured although the latter variables are not discussed further. A manipulation check (2 items, *r* = 0.87; e.g., “Korea (or, Korean people) seems to accept the apology for the past wrongdoings done by the Japanese people.”) was conducted at the end of the study, followed by attention checks (2 items; e.g., “According to this article, the Japanese company acknowledged the fact that Korean labors were forcibly taken in wartime.”). There were no participants who failed the attention checks. All items were rated on a 7-point scale, ranging from 1, *strongly disagree*, to 7, *strongly agree*. After completing all questionnaires, participants provided demographic information (gender, age, and nationality), made a guess about the study’s actual purpose, and then were debriefed. No participant suspected the study’s true intention.

### Results

#### Manipulation Check

Mean values for participants’ ratings on main variables across the three conditions are reported in Table 5. There was a significant condition effect on manipulation check, *F*(2, 206) = 98.15, *p* < 0.001, η^2^ = 0.49. Participants in the apology accepted condition perceived that Koreans expressed the acceptance of the apology significantly more compared to those in the apology rejected condition, *p* < 0.001, 95% CI (1.78, 2.72), and to those in the control condition, *p* < 0.001, 95% CI (2.04, 2.97), confirming that the manipulation was successful. There was no difference between the latter two conditions, *p* = 0.421, 95% CI (–0.74, 0.22).

**TABLE 5:** Means (standard deviations) of variables in the three experimental conditions (Study 2).

	Condition		
	Apology accepted	Apology rejected	Control
Manipulation check	4.80_a_ (1.27)	2.55_b_ (1.20)	2.29_b_ (1.06)
Perceived humanness of Koreans			
HN statements	4.30 (0.92)	4.14 (0.94)	4.12 (0.80)
HU statements	4.19_a_ (0.80)	3.78_b_ (0.99)	4.01_ab_ (0.93)
Relationship outcome measures			
Willingness to help	5.73 (1.00)	5.66 (1.41)	5.52 (1.72)
Willingness to apologize	5.00_ab_ (1.08)	5.23_a_ (1.15)	4.58_b_ (1.16)
Expectation of a future relationship	4.56_a_ (1.15)	3.86_b_ (1.27)	3.93_b_ (1.21)

*Mean values in a row with different subscripts are significantly different at **p** < 0.05 using Tukey’s HSD **post hoc** comparison, and no subscript means no significant differences between the conditions.*

#### Humanness Attributions

Human nature and HU traits showed unacceptable reliabilities (as = 0.53, 0.56, respectively) and were excluded in the current analysis as in Study 1. The Japanese participants’ attribution of humanness statements to Korean people was examined by two one-way ANOVAs on HN and HU. There was no significant effect of condition on HN, *F*(2, 206) = 0.88, *p* = 0.418. However, there was a significant condition effect on attributions of HU to Koreans, *F*(2, 206) = 3.547, *p* = 0.031, η^2^ = 0.03. *Post hoc* comparisons indicated that participants in the apology accepted condition attributed more HU to Koreans than those in the apology rejected condition, *p* = 0.028, 95% CI (0.03, 0.78). HU attribution to Koreans in the control condition did not significantly differ from that in the apology accepted condition, *p* = 0.69, 95% CI (–0.47, 0.19) and that in the apology rejected condition, *p* = 0.18, 95% CI (–0.04, 0.65). Thus, mirroring the findings in Study 1, these results are consistent with our hypothesis that Japanese participants would assign more HU, but not HN, to Koreans when the outgroup was described to be accepting (vs. rejecting) the ingroup’s apology.

#### Relationship Outcome Measures

Overall, participants, regardless of conditions, showed greater willingness to help and willingness to apologize than the middle point, *t*(208) = 13.71, *t*(208) = 5.43, respectively, both *ps* < 0.001. In contrast, future relationship expectation was significantly negative when the conditions were aggregated, *t*(207) = –4.26, *p* < 0.001. A comparison between conditions revealed that willingness to help was not significantly different across conditions, *F*(2, 206) = 0.56, *p* = 0.575. However, there were significant condition effects for willingness to apologize, *F*(2, 206) = 5.69, *p* = 0.004, η^2^ = 0.05, and for future relationship expectation, *F*(2, 205) = 7.40, *p* < 0.001, η^2^ = 0.07. A *post hoc*-test indicated that willingness to apologize was greater in the apology rejected condition, *p* = 0.003, 95% CI (0.18, 1.12), than in the control condition. There was no difference between the apology accepted condition and the control condition, *p* = 0.093, 95% CI (–0.05, 0.87), and between the apology accepted and apology rejected conditions, *p* = 0.653, 95% CI (–0.70, 0.23). The unexpected findings here may be partly because reminding participants that the outgroup did not accept the ingroup’s apology (vs. neutral) led to an even stronger will to apologize. However, it is still puzzling why the willingness to apologize was not different between apology accepted and rejected conditions. These findings may suggest that Japanese students’ willingness to apologize toward the victims is located higher than the neutral regardless of the outgroup’s acceptance. Future relationship was expected to be more positive in the apology accepted condition than in the apology rejected condition, *p* = 0.002, 95% CI (0.21, 1.20), and the control condition, *p* = 0.006, 95% CI (0.14, 1.12). There was no difference between the apology rejected condition and the control condition, *p* = 0.933, 95% CI (–0.58, 0.43).

#### Mediation Analysis

**FIGURE 4:**
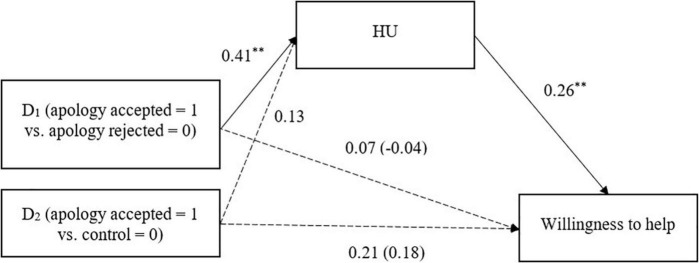
HU mediating the relationship between perceived apology acceptance and willingness to help (Study 2). Unstandardized coefficients are presented. Coefficients in parentheses denote direct effects after controlling for indirect effects. ^**^p < 0.01.

**FIGURE 5:**
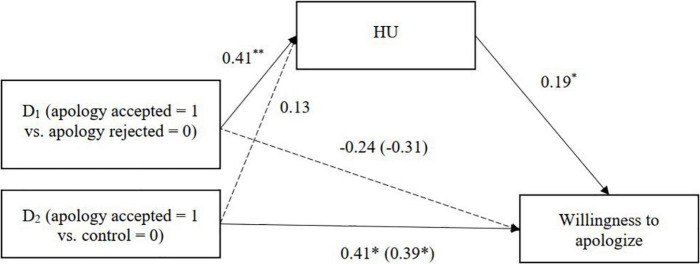
HU mediating the relationship between perceived apology acceptance and willingness to apologize (Study 2). Unstandardized coefficients are presented. Coefficients in parentheses denote direct effects after controlling for indirect effects. *p < 0.05, ^**^p < 0.01.

**FIGURE 6:**
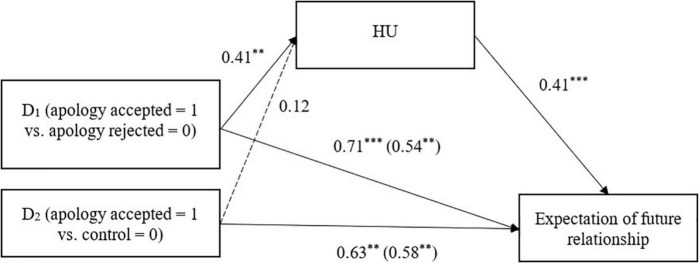
HU mediating the relationship between perceived apology acceptance and expectation of a future relationship (Study 2). Unstandardized coefficients are presented. Coefficients in parentheses denote direct effects after controlling for indirect effects. ^**^p < 0.01, ^***^p < 0.001.

We further examined whether attributions of HU mediated the relationship between perceived acceptance and the three relationship outcome variables. The process was identical to that in Study 1, but with HU as the mediating variable. The condition created two dummy variables: apology accepted vs. apology rejected conditions and apology accepted vs. control conditions (Table 6 and Figures 4–6). Japanese participants attributed more HU to Koreans when Japan’s apology was accepted (vs. rejected), and the HU attribution, in turn, was associated with stronger intentions to help Koreans and higher expectations of future intergroup relations.

**TABLE 6:** Indirect effects of condition on relationship outcome measures through human uniqueness (HU) (Study 2).

	Condition → HU → willingness to help	Condition → HU → willingness to apologize	Condition → HU → expectation of a future relationship
Apology accepted (1) vs. apology rejected (0)	0.11 [0.01, 0.24]	0.08 [–0.01, 0.19]	0.17 [0.04, 0.34]
Apology accepted (1) vs. control (0)	0.03 [–0.04, 0.13]	0.02 [–0.03, 0.11]	0.05 [–0.07, 0.19]

*Numbers indicate regression coefficients for indirect effects and respective 95% CIs.*

### Discussion

In Study 2, the Japanese participants ascribed more HU toward Koreans when the ingroup apology was perceived to be accepted (vs. rejected) by the outgroup. In the apology rejected condition, the Japanese participants presumably had more need to protect their moral image and justify past wrongdoings (Bandura, 1999; Castano and Giner-Sorolla, 2006), which resulted in an underestimation of Koreans’ uniquely human aspects. When the ingroup apology was accepted by Koreans, however, such need would decrease. These findings imply that the perpetrator’s attitudes toward the victimized are affected by the perceived reaction of the victim group to the ingroup’s attempt at reconciliation.

Different from findings in Study 1, relationship outcome measures (particularly, willingness to apologize and expectation of a future relationship) showed different patterns depending on perceived apology acceptance (vs. rejection). The outgroup’s acceptance of the apology led to more willingness to help and more optimistic views of a future relationship but not more willingness to apologize through HU. These findings imply the importance of humanness attribution of the outgroup for promoting intergroup relationships that may vary according to the perceived outgroup’s attitudes.

In addition, we observed an unanticipated pattern in the difference in willingness to apologize: participants in the apology rejected condition were more inclined to apologize than those in the control condition. It is possible that an article describing the forced labor issue in the apology rejected condition worked as a reminder and thus facilitated their willingness to apologize.

## General Discussion

Across two experimental studies, we investigated how members of two groups with an antagonistic history perceived each other’s humanness differently depending on the other’s intention for or against reconciliation. We expected that members of the victim group would assign more HN to the perpetrator group when the perpetrator group apologizes for their past wrongdoings and that the perpetrator group would see more HU in the victim group members if the victims accept their apologies. We tested these possibilities in two controlled studies using fictitious information with the historical background between Japan and Korea during the early twentieth century. In Study 1, Korean participants who perceived the Japanese people’s apologetic attitudes attributed more HN (i.e., less mechanistic dehumanization) to the outgroup compared to those who perceived the outgroup’s lack of apologetic action. In Study 2, Japanese participants who perceived Koreans’ positive attitudes toward the ingroup’s apology attributed more HU (i.e., less animalistic dehumanization) to the outgroup than those who perceived rejecting attitudes in the outgroup. An alternative interpretation is that apology (Study 1) and apology acceptance (Study 2) may have reduced dehumanization by increasing positive attitudes toward the outgroup. However, if this alone have happened, we would have observed significant reductions in both types of dehumanization. Because only the specific type of dehumanization was observed as we had predicted, we think that it is difficult to attribute the reduction in dehumanization only to the improvement of attitude toward the outgroup. To summarize, these symmetrical findings between the two groups suggest that humanness attribution of outgroup members depends on the outgroup’s actions and intentions with regard to mutual relations. Outgroup members may be dehumanized because of the ingroup’s needs (Castano and Giner-Sorolla, 2006; Leidner et al., 2013), but this dehumanization can be reduced by reconciliatory actions from the outgroup.

Although the effects of apology manipulation on relationship outcome measures were not significant among Korean participants in Study 1, it indirectly predicted all of the relationship outcome variables (willingness to help, willingness to forgive, and expectation of a future relationship) through the HN attribution. Similarly, in Study 2, apology acceptance manipulation indirectly predicted behavioral motivation (i.e., helping) as well as the expectation of future intergroup relationships through the perception of greater HU. In all, we demonstrated that apology by the perpetrator and acceptance by the victim can indirectly promote positive intergroup relations by leading the members of each group to recognize more humanness in the other. However, because of the limitations in non-experimental mediation analysis (Bullock et al., 2010; Imai et al., 2010; Fiedler et al., 2018), the mediation effects in this study should be interpreted with caution. Without experimentally manipulating mediator variables, it is improper to draw causal conclusions about the relationship between humanness and the dependent variables.

To the best of our knowledge, the current research is the first to experimentally address the effect of apology refusal and rejection on mutual dehumanization (cf., Tam et al., 2007). The current findings have significant theoretical implications in that they expand our understanding of intergroup apology, which can serve as a catalyst for perceiving humanness in others in the context of intergroup conflict. Although several lines of previous research investigated the effects of apology (Brown et al., 2008; Leonard et al., 2011) and forgiveness (Borris and Diehl, 1998; Cehajic et al., 2008; Hewstone et al., 2008) on reconciliation, few studies have looked at how dehumanization is involved in these exchanges. Specifically, we demonstrated the symmetrical mechanism of mutual dehumanization between the victim and perpetrator group members (i.e., perception of the victims’ HU and the perpetrators’ HN by each other) even in separate imaginary settings. Moreover, the effect of the issuance of apology or acceptance of apology by the outgroup has been rarely studied in this field, which adds another novelty to the current investigation. The overall results also have practical implications as they can be applied to other groups experiencing historical conflicts (e.g., Aboriginal Australians and European Australians).

Despite many implications, there are a few issues that call for future investigation. First, specificities in the control conditions in both studies introduce difficulty in interpreting the results. When we designed the experiments, we made the explicit decision to have control conditions that would show participants’ responses in their “default” state (i.e., what they would say in everyday life without any historical reminders) so that we could examine the manipulation effects in comparison to when virtually no information was provided. The control conditions are different from the two experimental conditions in two ways: They did not mention a past conflict between the two nations; they did not mention an apology being issued or not (in Study 1) or an apology being accepted or not (in Study 2). This introduces a confound because we cannot decisively say which of these two differences yielded differences with the experimental conditions. Because of this ambiguity with control conditions, we cannot be certain whether the issuance or acceptance of an apology led to less dehumanization or whether an apology refusal or rejection increased dehumanization. Conditions in which historical conflicts are presented without any mention of apology may enable a clearer interpretation. We speculate that Korean participants would attribute less humanness to the Japanese than when there is only neutral content (i.e., the current control condition) because they may assume that Japan is refusing to apologize if there is no mention of Japan’s apology (Lee, 2015). Similarly, if only past conflicts between the two countries are mentioned without information on whether Japan’s apology was accepted or not, Japanese participants may think that Korea refuses to accept the apology and may attribute less humanness to Koreans compared to the current control condition (although their attitude may be more ambivalent; Oh and Matsumoto, 2013). Such possibilities can be explored in future research.

Second, although we speculated a possible explanation (i.e., suppression effect) for the non-significant results of the apology effect, we did not directly verify it, which left a limitation in this study. Further research is needed to confirm how victims’ satisfactions (e.g., Philpot and Hornsey, 2008) and perceptions of sincerity (e.g., Berndsen et al., 2015; Chun, 2015) with the perpetrators’ apologies influence apology effect on outcome variables, and given that the current study was conducted with only university student samples, future research needs to replicate and expand our findings in more diverse populations to improve the generalizability.

According to the needs-based model of reconciliation (Shnabel and Nadler, 2008), the parties involved may have distinct needs as a result of coping with the unique threats they are facing: the needs to improve the power and status of the victim group and the needs to reduce the feelings of guilt for the perpetrator group (Castano and Giner-Sorolla, 2006). Therefore, the effects of the perceived outgroup’s response (i.e., apology or apology acceptance) on dehumanization could be related to the needs-based mechanisms, which are worth examining in future research. Also, it would be helpful to examine moderating or mediating effects of people’s group identification, as it affects antecedents and consequences of group-based guilt and apology for the past (Doosje et al., 2006, see also, Paladino et al., 2004; Castano and Giner-Sorolla, 2006). Collective victimhood of victim group members can also moderate the apology effect because a high level of collective victimhood is associated with more attributions of hostile intentions toward the perpetrator group (Schori-Eyal et al., 2017). If the victim’s collective victimhood is high, the victim will require a higher standard in apology, which may cause the apology effect to be negligible. On the other hand, because acknowledgment of collective victimization by the perpetrator group is an important factor in promoting intergroup reconciliation (Vollhardt, 2012), the victims’ perception of the perpetrators’ acknowledgment of collective victimization may influence the apology effect. Thus, the victim’s collective victimhood and perceived acknowledgments of collective victimization should be considered in future research on apology effects (e.g., Green et al., 2017). Additionally, Kteily et al. (2016) and Kteily and Bruneau (2017) suggested a novel antecedent of outgroup dehumanization: People who are aware that an outgroup dehumanizes them engage in dehumanizing the outgroup as a response, which in turn induces hostile attitudes to them. Combining this with our research model will further advance our understanding of intergroup apology and its consequences. For instance, investigating the possibility of the victims’ and perpetrators’ meta-dehumanization of each other as well as its moderation role between apology and forgiveness would be a fruitful avenue for future research. Finally, examining prosocial behaviors related to intergroup reconciliation beyond the intentions will benefit future studies.

## Conclusion

As an initial attempt to relate dehumanization with intergroup conflict and reconciliation with a special focus on apology issues, the current study demonstrates how perceived gestures toward or against reconciliation can influence humanness attribution of the outgroup. Supporting that different senses of humanness are denied in outgroup dehumanization, the study, based on real-life international issues, suggests the importance of understanding the outgroup’s attitudes and intentions in the dehumanization process, which in turn can affect the outlook of the future intergroup relations. The findings are expected to contribute to enlightening the process of intergroup reconciliation and facilitating more positive intergroup relations.

## Data Availability Statement

The raw data supporting the conclusions of this article will be made available by the authors, without undue reservation.

## Ethics Statement

The studies involving human participants were reviewed and approved by the Chungbuk National University Institutional Review Board, and Nagoya University of Commerce and Business Institutional Review Board. The patients/participants provided their written informed consent to participate in this study.

## Author Contributions

All authors conceived and conducted the studies, analyzed the data, and wrote the manuscript.

## Conflict of Interest

The authors declare that the research was conducted in the absence of any commercial or financial relationships that could be construed as a potential conflict of interest.

## Publisher’s Note

All claims expressed in this article are solely those of the authors and do not necessarily represent those of their affiliated organizations, or those of the publisher, the editors and the reviewers. Any product that may be evaluated in this article, or claim that may be made by its manufacturer, is not guaranteed or endorsed by the publisher.

## Footnotes

1 Apology is defined as an act whereby the perpetrator acknowledges responsibility for the past abuse and expresses emotions of regret or guilt (Darby and Schlenker, 1982; Tavuchis, 1991). Making restitution is also an important component of an apology (Schlenker and Darby, 1981; Schmitt et al., 2004). Based on these conceptualizations, an intergroup apology can be thought of as offending group members’ acts that include the acknowledgment of their ingroup’s past wrongdoings, and expression of guilt as well as of intention of reparation.

## References

[B1] Andrighetto L. Baldissarri C. Lattanzio S. Loughnan S. Volpato C. (2014). Human-itarian aid? Two forms of dehumanization and willingness to help after natural disasters. *Br. J. Soc. Psychol.* 53 573–584. 10.1111/bjso.12066 24588786

[B2] Bain P. Park J. Kwok C. Haslam N. (2009). Attributing human uniqueness and human nature to cultural groups: distinct forms of subtle dehumanization. *Group Proc. Intergr. Relat.* 12 789–805. 10.1177/1368430209340415

[B3] Bandura A. (1999). Moral disengagement in the perpetration of inhumanities. *Pers. Soc. Psychol. Rev.* 3 193–209. 10.1207/s15327957pspr0303_3 15661671

[B4] Bandura A. Underwood B. Fromson M. E. (1975). Disinhibition of aggression through diffusion of responsibility and dehumanization of victims. *J. Res. Pers.* 9 253–269. 10.1016/0092-6566(75)90001-X

[B5] Baron R. M. Kenny D. A. (1986). The moderator−mediator variable distinction in social psychological research: conceptual, strategic, and statistical considerations. *J. Pers. Soc. Psychol.* 51 1173–1182. 10.1037//0022-3514.51.6.1173 3806354

[B6] Bastian B. Haslam N. (2010). Excluded from humanity: the dehumanizing effects of social ostracism. *J. Exp. Soc. Psychol.* 46 107–113. 10.1016/j.jesp.2009.06.022

[B7] Bastian B. Haslam N. (2011). Experiencing dehumanization: cognitive and emotional effects of everyday dehumanization. *Basic Appl. Soc. Psychol.* 33 295–303. 10.1080/01973533.2011.614132

[B8] Bastian B. Jetten J. Radke H. R. M. (2012). Cyber-dehumanization: violent video game play diminishes our humanity. *J. Exp. Soc. Psychol.* 48 486–491. 10.1016/j.jesp.2011.10.009

[B9] Berndsen M. Hornsey M. J. Wohl M. J. (2015). The impact of a victim-focused apology on forgiveness in an intergroup context. *Group Process. Intergr. Relat.* 18 726–739. 10.1177/1368430215586275

[B10] Borinca I. Falomir-Pichastor J. M. Andrighetto L. Halabi S. (2021). Overcoming negative reactions to prosocial intergroup behaviors in post-conflict societies: the power of intergroup apology. *J. Exp. Soc. Psychol.* 95:104140. 10.1016/j.jesp.2021.104140

[B11] Borris E. R. Diehl P. F. (1998). “Forgiveness, reconciliation and the contribution of international peacekeeping,” in *The Psychology of Peacekeeping*, ed. Langholtz H. J. (New York, NY: Praeger), 207–222.

[B12] Boudjemadi V. Demoulin S. Bastart J. (2017). Animalistic dehumanization of older people by younger ones: variations of humanness perceptions as a function of a target’s age. *Psychol. Aging* 32 293–306. 10.1037/pag0000161 28230382

[B13] Branscombe N. R. Doosje B. McGarty C. (2002). “Antecedents and consequences of group-based guilt,” in *From Prejudice to Intergroup Emotions: Differentiated Reactions to Social Groups*, eds Mackie D. M. Smith E. R. (Philadelphia: Psychology Press), 49–66. 10.4324/9781315783000

[B14] Brown R. P. Wohl M. J. A. Exline J. J. (2008). Taking up offenses: secondhand forgiveness and group identification. *Pers. Soc. Psychol. Bull.* 34 1406–1419. 10.1177/0146167208321538 18768746

[B15] Bullock J. G. Green D. P. Ha S. E. (2010). Yes, but what’s the mechanism? (Don’t expect an easy answer). *J. Pers. Soc. Psychol.* 98 550–558. 10.1037/a0018933 20307128

[B16] Castano E. Giner-Sorolla R. (2006). Not quite human: infrahumanization in response to collective responsibility for intergroup killing. *J. Pers. Soc. Psychol.* 90 804–818. 10.1037/0022-3514.90.5.804 16737374

[B17] Cehajic S. Brown R. Castano E. (2008). Forgive and forget? Antecedents and consequences of intergroup forgiveness in Bosnia and Herzegovina. *Political Psychol.* 29 351–367. 10.1111/j.1467-9221.2008.00634.x

[B18] Chalk F. R. Jonassohn K. (1990). *The history and sociology of genocide: Analyses and case studies.* New Haven: Yale University Press, 10.1080/03612759.1991.9949515

[B19] Chun J. H. (2015). Beyond “Dissatisfaction” and “Apology Fatigue”: four types of Japanese official apology. *Pacific Focus* 30 249–269. 10.1111/pafo.12045

[B20] Cuddy A. J. C. Rock M. S. Norton M. I. (2007). Aid in the aftermath of Hurricane Katrina: inferences of secondary emotions and intergroup helping. *Group Process. Intergr. Relat.* 10 107–118. 10.1177/1368430207071344

[B21] Darby B. W. Schlenker B. R. (1982). Children’s reactions to apologies. *J. Pers. Soc. Psychol.* 43 742–753. 10.1037/0022-3514.43.4.742

[B22] Doosje B. E. J. Branscombe N. R. Spears R. Manstead A. S. (2006). Antecedents and consequences of group-based guilt: the effects of ingroup identification. *Group Process. Intergr. Relat.* 9 325–388. 10.1177/1368430206064637

[B23] Faul F. Erdfelder E. Lang A. G. Buchner A. (2007). G* Power 3: A flexible statistical power analysis program for the social, behavioral, and biomedical sciences. *Behav. Res. Methods* 39 175–191. 10.3758/BF03193146 17695343

[B24] Fiedler K. Harris C. Schott M. (2018). Unwarranted inferences from statistical mediation tests–An analysis of articles published in 2015. *J. Exp. Soc. Psychol.* 75 95–102. 10.1016/j.jesp.2017.11.008

[B25] Goff P. A. Eberhardt J. L. Williams M. J. Jackson M. C. (2008). Not yet human: implicit knowledge, historical dehumanization, and contemporary consequences. *J. Pers. Soc. Psychol.* 94 292–306. 10.1037/0022-3514.94.2.292 18211178

[B26] Green E. G. Visintin E. P. Hristova A. Bozhanova A. Pereira A. Staerklé C. (2017). Collective victimhood and acknowledgement of outgroup suffering across history: majority and minority perspectives. *Eur. J. Soc. Psychol.* 47 228–240. 10.1002/ejsp.2237

[B27] Greitemeyer T. McLatchie N. (2011). Denying humanness to others: a newly discovered mechanism by which violent video games increase aggressive behavior. *Psychol. Sci.* 22 659–665. 10.1177/0956797611403320 21422464

[B28] Haque O. S. Waytz A. (2012). Dehumanization in medicine: Causes, solutions, and functions. *Perspect. Psychol. Sci.* 7 176–186. 10.1177/1745691611429706 26168442

[B29] Harth N. S. Hornsey M. J. Barlow F. K. (2011). Emotional responses to rejection of gestures of intergroup reconciliation. *Pers. Soc. Psychol. Bull.* 37 815–829. 10.1177/0146167211400617 21402754

[B30] Haslam N. (2006). Dehumanization: an integrative review. *Pers. Soc. Psychol. Rev.* 10 252–264. 10.1207/s15327957pspr1003_4 16859440

[B31] Haslam N. Bain P. Douge L. Lee M. Bastian B. (2005). More human than you: Attributing humanness to self and others. *J. Pers. Soc. Psychol* 89:7. 7 10.1037/0022-3514.89.6.93 16393026

[B32] Haslam N. Loughnan S. (2014). Dehumanization and infrahumanization. *Annu. Rev. Psychol*. 65 399–423. 10.1146/annurev-psych-010213-115045 23808915

[B33] Hayashi H. (2008). Disputes in Japan over the Japanese military “Comfort Women” system and its perception in history. *Ann. Am. Acad. Political Soc. Sci.* 617 123–132. 10.1177/0002716208314191

[B34] Hayes A. F. (2009). Beyond Baron and Kenny: Statistical mediation analysis in the new millennium. *Commun. Monogr.* 76 408–420. 10.1080/03637750903310360

[B35] Hayes A. F. Preacher K. J. (2014). Statistical mediation analysis with a multicategorical independent variable. *Br. J. Math. Stat. Psychol.* 67 451–470. 10.1111/bmsp.12028 24188158

[B36] Hewstone M. Kenworthy J. B. Cairns E. Tausch N. Hughes J. Tam T. (2008). “Stepping stones to reconciliation in Northern Ireland: Intergroup contact, forgiveness, and trust,” in *The Social Psychology of Intergroup Reconciliation*, eds Nadler A. Malloy T. E. Fisher J. D. (Oxford: Oxford University Press), 199–226. 10.1093/acprof:oso/9780195300314.003.0010

[B37] Imai K. Keele L. Tingley D. (2010). A general approach to causal mediation analysis. *Psychol. Methods* 15 309–334. 10.1037/a0020761 20954780

[B38] Inquisit 4 (2015). *Computer software.* Available Online at: https://www.millisecond.com (accessed November 29, 2018).

[B39] Kelman H. C. (1976). “Violence without restraint: Reflections on the dehumanization of victims and victimizers,” in *Varieties of Psychohistory*, eds Kren G. M. Rappoport L. H. (New York, NY: Springer), 282–314. 10.1111/j.1540-4560.1973.tb00102.x

[B40] Kteily N. Bruneau E. (2017). Backlash: The politics and real-world consequences of minority group dehumanization. *Pers. Soc. Psychol. Bull.* 43 87–104. 10.1177/0146167216675334 28903649

[B41] Kteily N. Hodson G. Bruneau E. (2016). They see us as less than human: Metadehumanization predicts intergroup conflict via reciprocal dehumanization. *J. Pers. Soc. Psychol.* 110 343–370. 10.1037/pspa0000044 26963763

[B42] Lee S. H. (2015). Influx of Japanese popular culture and Korea-Japan relations. *Jap. Cult. Stud.* 53 273–294. 10.18075/jcs.53.201501.273

[B43] Leidner B. Castano E. Ginges J. (2013). Dehumanization, retributive and restorative justice, and aggressive versus diplomatic intergroup conflict resolution strategies. *Pers. Soc. Psychol. Bull*. 39 181–192. 10.1177/0146167212472208 23386655

[B44] Leonard D. J. Mackie D. M. Smith E. R. (2011). Emotional responses to intergroup apology mediate intergroup forgiveness and retribution. *J. Exp. Soc. Psychol.* 47 1198–1206. 10.1016/j.jesp.2011.05.002

[B45] Leyens J.-P. Paladino P. M. Rodriguez-Torres R. Vaes J. Demoulin S. Rodriguez-Perez A. (2000). The emotional side of prejudice: The attribution of secondary emotions to ingroups and outgroups. *Pers. Soc. Psychol. Rev.* 4 186–197. 10.1207/S15327957PSPR0402_06

[B46] Leyens J.-P. Rodriguez-Perez A. Rodriguez-Torres R. Gaunt R. Paladino M. P. Vaes J. (2001). Psychological essentialism and the differential attribution of uniquely human emotions to ingroups and outgroups. *Eur. J. Soc. Psychol.* 31 395–411. 10.1002/ejsp.50

[B47] Loughnan S. Haslam N. (2007). Animals and androids: implicit associations between social categories and nonhumans. *Psychol. Sci.* 18 116–121. 10.1111/j.1467-9280.2007.01858.x 17425529

[B48] MacKinnon D. P. Krull J. L. Lockwood C. M. (2000). Equivalence of the mediation, confounding and suppression effect. *Prev. Sci.* 1 173–181. 10.1023/A:1026595011371 11523746 PMC2819361

[B49] Maoz I. McCauley C. (2008). Threat, dehumanization, and support for retaliatory aggressive policies in asymmetric conflict. *J. Confl. Resolut.* 52 93–116. 10.1177/0022002707308597

[B50] Morera M. D. Quiles M. N. Correa A. D. Delgado N. Leyens J.-P. (2018). Perception of mind and dehumanization: Human, animal, or machine? *Int. J. Psychol.* 53 253–260. 10.1002/ijop.12375 27480887

[B51] Oh D.-Y. Shin C.-W. (2010). Korean, Japanese attitudes about each other growing closer: survey. *Korea JoongAng Daily* 2010:2925061.

[B52] Oh J.-B. Matsumoto A. (2013). Qualitative research on the images of Korean people among Japanese college students. *Papers Ling. Sci.* 17 59–72. 10.1002/(sici)1098-108x(199803)23:2<153::aid-eat5>3.0.co;2-j 9503240

[B53] Pacilli M. G. Roccato M. Pagliaro S. Russo S. (2016). From political opponents to enemies? The role of perceived moral distance in the animalistic dehumanization of the political outgroup. *Group Process. Intergr. Relat.* 19 360–373. 10.1177/1368430215590490

[B54] Paladino M. P. Vaes J. Castano E. Demoulin S. Leyens J.-P. (2004). Emotional infra-humanization in intergroup relations: the role of national identification in the attribution of primary and secondary emotions to Italians and Germans. *Curr. Psychol. Cogn.* 22 519–536.

[B55] Park J. Haslam N. Kashima Y. (2012). Relational to the core: lay theories of humanness in Australia, Japan, and Korea. *J. Cross-Cult. Psychol.* 43 774–783. 10.1177/0022022111414417

[B56] Park Y. O. Park S. H. (2015). Observing social exclusion leads to dehumanizing the victim. *Front. Psychol*. 6:1815. 10.3389/fpsyg.2015.01815 26635705 PMC4656819

[B57] Philpot C. R. Hornsey M. J. (2008). What happens when groups say sorry: the effect of intergroup apologies on their recipients. *Pers. Soc. Psychol. Bull*. 34 474–487. 10.1177/0146167207311283 18340033

[B58] Philpot C. R. Hornsey M. J. (2011). Memory for intergroup apologies and its relationship with forgiveness. *Eur. J. Soc. Psychol.* 41 96–106. 10.1002/ejsp.741

[B59] Piper N. (2001). Transnational women’s activism in Japan and Korea: the unresolved issue of military sexual slavery. *Glob. Netw.* 1 155–170. 10.1111/1471-0374.00010

[B60] Rucker D. D. Preacher K. J. Tormala Z. L. Petty R. E. (2011). Mediation analysis in social psychology: Current practices and new recommendations. *Soc. Personal. Psychol. Compass* 5 359–371. 10.1111/j.1751-9004.2011.00355.x

[B61] Schlenker B. R. Darby B. W. (1981). The use of apologies in social predicaments. *Soc. Psychol. Q.* 44 271–278. 10.2307/3033840

[B62] Schmitt M. Gollwitzer M. Förster N. Montada L. (2004). Effects of objective and subjective account components on forgiving. *J. Soc. Psychol.* 144 465–486. 10.3200/SOCP.144.5.465-486 15449697

[B63] Schori-Eyal N. Klar Y. Ben-Ami Y. (2017). Perpetual ingroup victimhood as a distorted lens: effects on attribution and categorization. *Eur. J. Soc. Psychol.* 47 180–194. 10.1002/ejsp.2250

[B64] Shibata R. (2018). “Apology and Forgiveness in East Asia,” in *Identity, Trust, and Reconciliation in East Asia*, ed. Clements K. (Cham: Palgrave Macmillan), 271–297. 10.1007/978-3-319-54897-5_12

[B65] Shnabel N. Nadler A. (2008). A needs-based model of reconciliation: satisfying the differential emotional needs of victim and perpetrator as a key to promoting reconciliation. *J. Pers. Soc. Psychol*. 94 116–132. 10.1037/0022-3514.94.1.116 18179322

[B66] Shnabel N. Nadler A. Ullrich J. Dovidio J. F. Carmi D. (2009). Promoting reconciliation through the satisfaction of the emotional needs of victimized and perpetrating group members: the needs-based model of reconciliation. *Pers. Soc. Psychol. Bull*. 35 1021–1030. 10.1177/0146167209336610 19498070

[B67] Soble J. (2013). *‘Comfort women’ gaffe wounds Osaka mayor. Financial Times.* Available Online at: https://www.ft.com/content/2b0241cc-c6c9-11e2-8a36-00144feab7de (accessed date 2013, May 27)

[B68] Tam T. Hewstone M. Cairns E. Tausch N. Maio G. Kenworthy J. (2007). The impact of intergroup emotions on forgiveness in Northern Ireland. *Group Process. Intergr. Relat.* 10 119–136. 10.1177/1368430207071345

[B69] Tavuchis N. (1991). *Mea culpa: A sociology of apology and reconciliation.* Stanford, CA: Stanford University Press.

[B70] Vaes J. Bastian B. (2021). Tethered humanity: humanizing self and others in response to interpersonal harm. *Eur. J. Soc. Psychol.* 51 377–392. 10.1002/ejsp.2744

[B71] Vaes J. Paladino M. P. Castelli L. Leyens J.-P. Giovanazzi A. (2003). On the behavioral consequences of infrahumanization: the implicit role of uniquely human emotions in intergroup relations. *J. Pers. Soc. Psychol.* 85 1016–1034. 10.1037/0022-3514.85.6.1016 14674811

[B72] Viki G. T. Fullerton I. Raggett H. Tait F. Wiltshire S. (2012). The role of dehumanization in attitudes toward the social exclusion and rehabilitation of sex offenders. *J. Appl. Soc. Psychol.* 42 2349–2367. 10.1111/j.1559-1816.2012.00944.x

[B73] Viki G. T. Osgood D. Phillips S. (2013). Dehumanization and self-reported proclivity to torture prisoners of war. *J. Exp. Soc. Psychol.* 49 325–328. 10.1016/j.jesp.2012.11.006

[B74] Vollhardt J. R. (2012). “Collective victimization,” in *The Oxford Handbook of Intergroup Conflict*, ed. Tropp L. R. (New York, NY: Oxford University Press), 136–157. 10.1093/oxfordhb/9780199747672.001.0001

[B75] Waytz A. Epley N. (2012). Social connection enables dehumanization. *J. Exp. Soc. Psychol.* 48 70–76. 10.1016/j.jesp.2011.07.012

[B76] Wenzel M. Anvari F. de Vel-Palumbo M. Bury S. M. (2017). Collective apology, hope, and forgiveness. *J. Exp. Soc. Psychol.* 72 75–87. 10.1016/j.jesp.2017.05.003

[B77] Wenzel M. Okimoto T. G. (2015). “We forgive”: a group’s act of forgiveness and its restorative effects on members’ feelings of justice and sentiments towards the offender group. *Group Process. Intergr. Relat.* 18 655–675. 10.1177/1368430215586274

[B78] Wohl M. J. Hornsey M. J. Bennett S. H. (2012). Why group apologies succeed and fail: intergroup forgiveness and the role of primary and secondary emotions. *J. Pers. Soc. Psychol*. 102 306–322. 10.1037/a0024838 21806306

[B79] Yang W. Jin S. He S. Fan Q. Zhu Y. (2015). The impact of power on humanity: Self-dehumanization in powerlessness. *PLoS One* 10:e0125721. 10.1371/journal.pone.0125721 26020267 PMC4447388

[B80] Zhao X. Lynch J. G. Jr. Chen Q. (2010). Reconsidering Baron and Kenny: Myths and truths about mediation analysis. *J. Consum. Res.* 37 197–206. 10.1086/651257

