# Supplementary material for: Apology and Its Acceptance: Perceived Reconciliatory Attitudes Reduce Outgroup Dehumanization
Source: Front Psychol. 2022 Apr 25;13:809513. doi: 10.3389/fpsyg.2022.809513 (PMC9083360; doi:10.3389/fpsyg.2022.809513)
Supplement: Supplementary file 1 [file Table_1.docx]

Supplementary Material

**Table 1.** List of Items for Measured Variables

| 1. HN statements |
| --- |
| *high HN*  I can feel warmth in Koreans/Japanese.  I think Koreans/Japanese are open minded and can think clearly.  I think Koreans/Japanese are emotional, responsive; and warm.  *low HN*  It seems like Koreans/Japanese have no depth. (reverse coded)  Koreans/Japanese seem like objects, not humans. (reverse coded)  Koreans/Japanese feel mechanical and cold, like robots. (reverse coded) |
| 2. HU statements |
| *high HU*  I think Koreans/Japanese are refined and cultured.  Koreans/Japanese seem like adults, not children.  Koreans/Japanese seem to have self-restraint.  I think Koreans/Japanese are intelligent, rational, and logical.  *low HU*  Koreans/Japanese seem like less than humans, like animals. (reverse coded)  I think Koreans/Japanese are unsophisticated. (reverse coded) |
| 3. willingness to help |
| If many people in Japan/Korea were to die because of an earthquake, we should help them.  If Japan/Korea suffered a terrorist attack and many people died, we should help them.  If Japan/Korea suffered from food shortage due to drought, we should help them. |
| 4. willingness to forgive (Study 1) |
| Koreans may be able to forgive the wrongdoings that Japan has perpetrated in the past.  Negative sentiments that Koreans have toward Japan originating from Japan’s past crimes may decrease.  Koreans may be able to shake off the negative emotions toward the crimes committed by Japan in the past. |
| 5. willingness to apologize (Study 2) |
| I think Japan had some damage to Korea in the first half of the 20th century.  Japan could apologize if it had done something wrong with Korea in the past.  When I look back, I think there are some regrets about the actions that Japan took against Korea and Koreans in the first half of the 20th century. |
| 6. expectation of relationship |
| The relationship between Korea and Japan will be better than now.  Korea and Japan will cooperate more for future progress.  Korea and Japan will have more mutual exchange.  Korea and Japan will have more friendly attitude toward each other. |
| 7. manipulation check  (Study 1) |
| Japan seems to have acknowledged its past wrongdoings against Korea.  Japan is expressing apologies for the comfort women. |
| (Study 2)  Korea (or, Korean people) seems to accept the apology for the past wrongdoings done by the Japanese people.  Korea recently accepted Japanese company’s apology for the forced Korean labors in wartime. |
| 8. attention check  (Study 1) |
| According to this article, Japanese government acknowledged the forced recruitment of comfort women.  According to this article, Japanese government apologized to comfort women victims.  (Study 2)  According to this article, the Japanese company acknowledged the fact that Korean labors were forcibly taken in wartime.  According to this article, the Japanese company has been apologizing to the forced Korean workers since the war ended. |
